# Correlation of *RET* somatic mutations with clinicopathological features in sporadic medullary thyroid carcinomas

**DOI:** 10.1038/sj.bjc.6605056

**Published:** 2009-04-28

**Authors:** M M Moura, B M Cavaco, A E Pinto, R Domingues, J R Santos, M O Cid, M J Bugalho, V Leite

**Affiliations:** 1 Centro de Investigação de Patobiologia Molecular (CIPM), Instituto Português de Oncologia de Lisboa Francisco Gentil E.P.E., Rua Prof. Lima Basto, 1099-023 Lisboa, Portugal; 2 Serviço de Anatomia Patológica, Instituto Português de Oncologia de Lisboa Francisco Gentil E.P.E., Rua Prof. Lima Basto, 1099-023 Lisboa, Portugal; 3 Serviço de Cirurgia Cabeça e Pescoço, Instituto Português de Oncologia de Lisboa Francisco Gentil E.P.E., Rua Prof. Lima Basto, 1099-023 Lisboa, Portugal; 4 Serviço de Endocrinologia, Instituto Português de Oncologia de Lisboa Francisco Gentil E.P.E., Rua Prof. Lima Basto, 1099-023 Lisboa, Portugal; 5 Faculdade de Ciências Médicas, Universidade Nova de Lisboa, Campo Mártires da Pátria 130, 1150-228 Lisboa, Portugal

**Keywords:** sporadic medullary thyroid carcinoma, *RET* proto-oncogene, somatic mutation, genetic screening, prognosis

## Abstract

Screening of *RE*arranged during *T*ransfection (*RET*) gene mutations has been carried out in different series of sporadic medullary thyroid carcinomas (MTC). *RET*-positive tumours seem to be associated to a worse clinical outcome. However, the correlation between the type of *RET* mutation and the patients' clinicopathological data has not been evaluated yet.

We analysed *RET* exons 5, 8, 10–16 in fifty-one sporadic MTC, and found somatic mutations in thirty-three (64.7%) tumours. Among the *RET*-positive cases, exon 16 was the most frequently affected (60.6%). Two novel somatic mutations (Cys630Gly, c.1881del18) were identified. MTC patients were divided into three groups: group 1, with mutations in *RET* exons 15 and 16; group 2, with other *RET* mutations; group 3, having no *RET* mutations. Group 1 had higher prevalence (*P*=0.0051) and number of lymph node metastases (*P*=0.0017), and presented more often multifocal tumours (*P*=0.037) and persistent disease at last control (*P*=0.0242) than group 2. Detectable serum calcitonin levels at last screening (*P*=0.0119) and stage IV disease (*P*=0.0145) were more frequent in group 1, than in the other groups.

Our results suggest that, among the sporadic MTC, cases with *RET* mutations in exons 15 and 16 are associated with the worst prognosis. Cases with other *RET* mutations have the most indolent course, and those with no *RET* mutations have an intermediate risk.

Medullary thyroid carcinoma (MTC) is a rare tumour that represents 5–10% of all types of thyroid cancer, and accounts for a disproportionate number of thyroid cancer deaths (Hundahl *et al*, 1998). Except for surgery, therapy for MTC is generally ineffective. MTC may occur sporadically (in about 75% of cases) or as a part of the autosomal dominantly inherited cancer syndrome, known as multiple endocrine neoplasia type 2 (MEN 2) (Mulligan *et al*, 1993; Eng, 1999; Frank-Raue *et al*, 2007). MTC is the most common cause of death in patients with MEN 2 (Skinner *et al*, 2005). This familial type of thyroid carcinoma usually originates as multifocal C-cell hyperplasia, its progression to MTC is extremely variable, and may take several years (Carling, 2005). In sporadic cases, the mean age at presentation is 50 years, with a slight female predominance (Matias-Guiu *et al*, 2004).

Activating germline mutations in the *RE*arranged during *T*ransfection (*RET*) gene are detected in over 95% of MEN 2 cases (Mulligan *et al*, 1993; Marx, 2005). The oncogenic potential of different *RET* mutations seems to be dependent on the site of the amino acid change, and may account for the diverse phenotypes observed in MEN 2 patients (Asai *et al*, 1995).

The screening of *RET* mutations has been carried out in different series of sporadic MTC, however the observed frequencies are variable (12–100%) (Hofstra *et al*, 1994; Zedenius *et al*, 1994; Jhiang *et al*, 1996; Marsh *et al*, 1996, 2003; Romei *et al*, 1996; Wohllk *et al*, 1996; Bugalho *et al*, 1997; Scurini *et al*, 1998; Shan *et al*, 1998; Uchino *et al*, 1998, 1999; Bockhorn *et al*, 1999; Dvorakova *et al*, 2008; Elisei *et al*, 2008). Met918Thr *RET* mutation is the most common somatic mutation in sporadic forms of MTC, and its detection rate varies greatly (5–66%) in the published literature (Zedenius *et al*, 1994; Marsh *et al*, 1996; Romei *et al*, 1996; Wohllk *et al*, 1996; Bugalho *et al*, 1997; Scurini *et al*, 1998; Uchino *et al*, 1998, 1999; Dvorakova *et al*, 2008; Elisei *et al*, 2008). However, in some of these studies, the authors have screened sporadic MTC for only a few specific mutations, mostly in codon 918 (Hofstra *et al*, 1994; Shan *et al*, 1998; Bockhorn *et al*, 1999; Marsh *et al*, 2003). Therefore, the number of exons screened, as well as the sizes of the analysed series, may explain some of the reported differences in the prevalence of *RET* mutations in sporadic MTC. In addition, ethnic or environmental factors, differences in detection or in sampling methods may also account for the reported differences (Uchino *et al*, 1998; Dvorakova *et al*, 2008). In some cohorts, besides the Met918Thr mutation, other somatic mutations were also detected, at a lower frequency in exons 10, 11, 12, 13 and 15 (Bugalho *et al*, 1997; Scurini *et al*, 1998; Uchino *et al*, 1999).

The major somatic mutation (Met918Thr) localised in the tyrosine kinase domain in exon 16 of *RET* (Marini *et al*, 2006) has been associated to a worse clinical outcome in sporadic MTC when compared with tumours that did not harbour this mutation (Zedenius *et al*, 1994, 1995; Wohllk *et al*, 1996; Schilling *et al*, 2001).

Several reports have presented contradictory results concerning the ploidy pattern in MTC. Schröder *et al* (1988) found that most MTC have a diploid DNA pattern, and that a benign disease course was twice as frequent in patients with diploid tumours compared with aneuploid tumours. Conversely, the results presented by Lindsay (1970) seemed to be more consistent with aneuploidy in MTC.

In this study, we carried out a comprehensive analysis of exons 5, 8 and 10–16 of *RET* to evaluate the prevalence of somatic mutations in a series of fifty-one sporadic MTC and to correlate with clinicopathological characteristics of the patients, including tumour ploidy pattern.

## Materials and methods

### Patients

A total of fifty-two unrelated patients with MTC without family history of the disease were studied for *RET* mutations. A detailed personal history was obtained from all patients. All individuals were of Caucasian origin (34 females and 18 males). Each patient underwent total thyroidectomy, with the exception of two patients who were submitted to partial thyroidectomy. The diagnosis of MTC was confirmed by histopathology of the surgically removed tumours. The Tumour-Node-Metastases (TNM) classification of all tumour specimens was carried out after the criteria described in the WHO classification of thyroid tumours (DeLellis *et al*, 2004). Stage grouping was addressed according to the TNM classification (Sobin and Wittekind, 2002), namely, stage I (T1N0M0), stage II (T2N0M0), stage III (T3N0M0 or T1-T3N1aM0) and stage IV (T1-T3N1bM0, T4N0-N1M0 or T1-T4N0-N1M1).

The number of truly sporadic MTC patients was reduced to fifty-one, as a germline mutation was found in one case.

Eight of the fifty-one cases (Table 1, patients 10, 15, 34, 35, 36, 38, 43 and 45) were earlier published by our group (Bugalho *et al*, 1997, 2000).

This study was carried out following guidelines approved by the local institution ethical board.

### DNA flow cytometry

DNA flow cytometry analysis was carried out on paraffin-embedded material, according to the method of Hedley *et al* (1983), with slight modifications (André *et al*, 2007).

### Serum calcitonin measurements

Serum calcitonin (CT) levels were determined using a solid-phase, enzyme-labelled, two-site chemiluminescent immunoenzymatic assay (Immulite 2000 Calcitonin, Siemens Medical Solutions Diagnostic Ltd., Llanberis, Gwynedd, UK) with the Immulite 2000 Automated Analyser (Siemens Medical Solutions Diagnostic Ltd.). CT values <2.0 ng l^–1^ were regarded as undetectable.

### *RET* variant analysis

DNA was extracted from tumour samples frozen in liquid nitrogen (*n*=47) following standard protocols. Otherwise, DNA was isolated from formalin-fixed paraffin-embedded tumour tissues (*n*=5), as described earlier (Imyanitov *et al*, 2001). Exons 5, 8 and 10 through 16 of *RET* were amplified by PCR. Sequences of the oligonucleotide primers and amplification conditions are available on request. Sequencing was carried out in both sense and antisense directions, using the same primers as for PCR amplification and the ABI PRISM BigDye Terminator version 1.1 Cycle Sequencing Kit (Applied Biosystems, Foster City, CA, USA), in an automated DNA sequencer (ABI PRISM 310 Genetic Analyser, Applied Biosystems). All the mutations identified were confirmed by two independent experiments (restriction enzyme analysis, or repeated sequence analysis). To support somatic origin of the mutations, constitutional DNA from peripheral blood or non-tumourous tissue from the same patient was also analysed.

### Statistical analysis

The statistical analysis was accomplished using GraphPad Prism version 4.0 statistical software (GraphPad Software Inc., San Diego, CA, USA) and SPSS 13.0 for Windows (SPSS Inc., Chicago, IL, USA). Values were expressed as mean±s.e.m. The *χ*^2^ or Fisher's exact tests, and one-way analysis of variance or Kruskal–Wallis test were used according to the studied variables. Survival curves were analysed using the Kaplan–Meier method, and the statistical significance was assessed by the logrank test. Values of *P*<0.05 were considered statistically significant.

## Results

### Genetic analysis

One out of the fifty-two (1.9%) cases of clinically apparently sporadic MTC carried a new germline mutation (Cys515Trp) located in exon 8 (manuscript under preparation). This case was excluded from further analysis.

In the remaining fifty-one sporadic MTC cases, thirty-three (64.7%) had mutations in *RET* exons 10, 11, 15 and 16 (Table 2). The absence of these mutations in the constitutional DNA excluded a germline origin.

Among the *RET*-positive cases, exon 16 was the most frequently affected (60.6%) by the same specific Met918Thr mutation, followed by exon 11 (21.2%). *RET* mutations were also detected in exons 10 (9.1%) and 15 (9.1%). In the present series, two novel *RET* mutations (Cys630Gly and c.1881del18) located at exon 11 were identified. The novel Cys630Gly variant creates a restriction site for the enzyme *Bsr*I, facilitating its independent confirmation (data not shown). The other novel variant (c.1881del18) is expected to lead to the replacement of seven amino acid residues by a glutamic acid residue. In this case, PCR amplification originated two fragments: the expected wild-type product (322 bp) and a smaller mutant product (304 bp), allowing the independent sequencing of both alleles (data not shown). Together with this mutation, and in the same allele, an unreported heterozygous nucleotide change in codon 634 (TGC to TGT) that does not predict an amino acid alteration (Cys to Cys) was found (data not shown).

In the MTC tissue of patient 51, with a Cys618Arg mutation (Table 1), the wild-type allele was not detected. The finding of allelic loss at flanking markers D10S141 and ZNF22 showed hemizygosity for this mutation (data not shown).

No mutations were identified in the other analysed exons, namely, exons 5, 8, 12, 13 or 14 (Table 2).

Forty-four (86.3%) tumours displayed a diploid DNA content and seven (13.7%) were aneuploid. No correlation between the presence or type of *RET* mutation and the ploidy pattern was observed (Table 3).

### Clinical evaluation

Table 1 describes the clinical and pathological data of the fifty-one patients with sporadic MTC.

In the thirty-three MTC patients (19 females and 14 males) carrying somatic *RET* mutations, the mean age at surgery and mean follow-up time were 52.9 years (median 55, range 26–71) and 94.8 months (median 100, range 4–303), respectively. Lymph node and distant metastases were present in 23/33 (69.7%) and 11/31 (35.5%) cases, respectively. According to the TNM classification, four patients (12.1%) had stage I disease, three (9.1%) had stage II, two (6.1%) had stage III and twenty-four (72.7%) had stage IV. At the time of the last clinical screening, nine patients (29.0%) were free of disease, and twenty-two (71.0%) were non-disease free (seven of them were deceased from MTC). The status at last control from two patients was not available. Among the twenty-two patients with persistent disease, ten (47.6%) showed a biochemical persistence of the disease with detectable levels of serum CT, but no evidence of distant metastases, whereas eleven patients (52.4%) were affected by metastatic disease. Clinical data from one case were not available.

As regard to the eighteen MTC patients (14 females and 4 males) without somatic *RET* mutation, the mean age at surgery and mean follow-up were 55.8 years (median 59.5, range 27–82) and 90.8 months (median 73.5, range 0.6–252), respectively. Lymph node and distant metastases were present in 11/18 (61.1%) and 3/18 (16.7%) cases, respectively. Three patients (16.7%) had stage I disease, four (22.2%) had stage II, one (5.6%) had stage III and ten (55.6%) had stage IV, at last control. At the time of the last clinical screening, eight patients (44.4%) were free of disease and ten (55.6%) were non-disease free (two of them were deceased from MTC). Among the ten patients with persistent disease, six (66.7%) showed a biochemical persistence of the disease but no evidence of distant metastases, whereas three patients (33.3%) were affected by metastatic disease. Biochemical data from one case were not available.

For all these clinical and pathological data, there was no statistically significant difference between *RET*-positive and *RET*-negative patients. When the survival curves of *RET*-positive and *RET*-negative MTC patients were compared, a lower percentage of surviving patients was observed in the group with the somatic *RET* mutation, although not statistically significant (data not shown).

As the more aggressive *RET* mutations are in codons 883 and 918, which are classified as level 3 (de Groot *et al*, 2006), we compared clinical data of somatic Met918Thr and Ala883Phe *RET* mutation cases (group 1; *n*=23, 45.1%) *vs* cases carrying other somatic *RET* mutations (group 2; *n*=10, 19.6%), or having no *RET* mutation (group 3; *n*=18, 35.3%) (Table 3).

Group 1 cases (compared with group 2) had higher prevalence of lymph node metastases (*P*=0.0051), higher number of positive lymph nodes (*P*=0.0017), were more frequently multifocal (*P*=0.037) and had more often persistent disease at last control (*P*=0.0242). Moreover, group 1 was more frequently associated with detectable serum CT levels at the last screening (*P*=0.0119), as well as with stage IV (*P*=0.0145; *vs* stages I–III), than groups 2 and 3.

Serum CT levels at the last control were highest in group 1 (37521±13818 ng l^–1^), intermediate in group 3 (11501±6028 ng l^–1^) and lowest in group 2 (651.2±624.7 ng l^–1^). However, the difference was only statistically significant between group 1 and group 2 patients (Kruskal–Wallis test plus Dunn's multiple comparison test, *P*=0.0035). Also, the serum CT levels after surgery were higher in group 1 than in the other groups, but the difference did not reach statistical significance (group 1: 8096±3517 ng l^–1^, group 3: 1135±856.5 ng l^–1^, group 2: 206.4±132.2 ng l^–1^; Kruskal–Wallis test, *P*=0.0628).

Although not statistically significant, there was a trend for a higher prevalence of distant metastases in group 1.

There was no statistical significant difference between patients of the different groups regarding the remaining clinicopathological characteristics (Table 3). Furthermore, when the survival curves of MTC patients from the three groups were evaluated, no significant differences were observed between each group (data not shown).

## Discussion

Medullary thyroid carcinoma is clinically diagnosed as sporadic when the patient does not present other endocrine tumours, and when no other cases of MTC, pheochromocytoma or parathyroid disease are identified in the patient's family. However, only the exclusion of germline mutations in the *RET* proto-oncogene allows a definitive diagnosis of sporadic MTC.

The herein reported cohort is one of the largest single-country studies. Fifty-one sporadic MTC were analysed and somatic mutations were found in thirty-three (64.7%) cases. Two novel mutations were identified in exon 11 of the *RET* proto-oncogene, in two sporadic MTC cases: a heterozygous point mutation at codon 630 (Cys630Gly), and a 18 bp deletion at nucleotide c.1881 associated in the same allele with a silent nucleotide substitution at codon 634 (Cys634Cys). Both mutations are located in the cysteine-rich domain coding sequence, which, when mutated, has been shown to constitutively activate RET (Santoro *et al*, 1995, 2002).

In this study, eight earlier described missense changes in *RET* were detected in exons 10, 11, 15 and 16. In accordance with other studies, the most common mutation was Met918Thr with a detection rate of 39.2%, which represents 60.6% of all detected mutations. In one case, loss of heterozygosity at *RET* flanking microsatellite markers was detected, showing hemizygosity for a sporadic missense mutation (Cys618Arg), a rare genetic event that has been reported earlier for other *RET* mutations (Jindrichova *et al*, 2003; Dvoráková *et al*, 2006).

As *RET* mutations other than Met918Thr are rare, most of the reported series did not compare the clinicopathological characteristics of Met918Thr *vs* other *RET* mutations (Dvorakova *et al*, 2008; Elisei *et al*, 2008). In our study, 13/51 (25.5%) cases had a *RET* mutation other than Met918Thr, which allowed such comparison. On the basis of the recent literature, *RET* mutations have been stratified into three risk levels, regarding the predisposition to originate MTC, as well as their *in vitro* transforming activity. Patients with germline mutations in *RET* codons 883 (exon 15) and 918 (exon 16), for which thyroidectomy is recommended at an early age, have the highest risk for the early development and the most aggressive MTC growth (Evans *et al*, 2007). Likewise, these two mutations (which are considered as level 3) have the highest *in vitro* transforming activity. Therefore, in this study, cases with Ala883Phe or Met918Thr mutations were analysed in the same group (group 1), and compared with cases with other somatic *RET* mutations (group 2) and cases with no *RET* mutation (group 3).

A statistically significant correlation was shown between group 1 and the presence of lymph node metastases, as well as the number of positive lymph nodes at the time of surgery, multifocality and a non-disease free status, compared with group 2. This correlation may account for the significantly higher frequency of patients from group 1 in stage IV and with detectable serum CT level at last control, compared with groups 2 and 3, and also for the significantly increased levels of serum CT at the last control in group 1 cases in comparison with group 2. Furthermore, such correlation supports the hypothesis that mutations in *RET* exons 15 and 16 are related to a more aggressive behaviour of MTC. This could be explained by an earlier dissemination of Met918Thr and Ala883Phe cases to lymph nodes (Table 3). Indeed, 26.1% of MTC cases came to clinical attention because of lymph nodes in group 1, whereas this occurred in only 0.0% and 11.8% of the cases in groups 2 and 3, respectively.

The results presented in Table 3 show a trend towards the stratification of the three groups of sporadic MTC patients into risk levels on the basis of the statistically significant clinicopathological characteristics. Group 1 patients are at the highest risk for aggressive MTC, followed by group 3 at intermediate risk, and group 2 patients, which present the lowest risk for a worse clinical outcome. Therefore, our study shows that *RET* mutations in exons 15 and 16 are associated with a more aggressive behaviour of sporadic MTC than other *RET* mutations, as it has been shown *in vitro*, as well as in the hereditary variants of MTC.

Taken together, these results suggest that the screening of *RET* somatic mutations may be helpful in the management of patients with MTC, according to the presence and type of *RET* somatic mutation.

## Figures and Tables

**Table 1:** Clinical and genetic findings in fifty-one patients with sporadic MTC

**Patient no.**	**Sex**	***RET* somatic mutation**	**MTC presentation**	**Age at surgery (years)**	**Tumour size^a^ (cm)**	**Post-oper. serum CT (ng l^–1^)**	**Serum CT at last control (ng l^–1^)**	**TNM classification at last control**	**No. of positive lymph nodes**	**Overall survival (months)**	**Disease free survival (months)**	**Status at last control**	**Extragl. ext.**	**Vasc. inv.**	**Multif.**	**Ploidy pattern**
1	F	**c.1881del18+ Cys634Cys**	TN	50	1.5	19.4	132.0	T1N1bM0	5	149	0	NDF	No	NA	No	D
2	F	Neg	TN	50	2	Undetect.	Undetect.	T1N0M0	0	172	172	DF	No	No	No	D
3	M	**Cys630Gly**	TN	71	6	1290.0	5647.0	T4aN0MX	0	74	0	NDF	Yes	Yes	No	D
4	M	Neg	TN	69	12	449.8	53341.0	T4bN1aM0	2	196	0	NDF	Yes	Yes	No	D
5	M	Met918Thr	TN+LN	69	7	1610.0	14594.0	T4bmN1bM1	19	Dec/9	0	NDF	Yes	Yes	Yes	D
6	F	Met918Thr	TN+LN	59	3.5	869.0	16209.0	T2N1bM1	7	167	0	NDF	No	NA	No	D
7	F	Met918Thr	TN	39	5	2.2	9361.0	T3N1bM0	20	303	24	NDF	NA	NA	No	D
8	F	Neg	LN	77	3.5	NA	NA	T4aN1bM0	18	18 days	0	NDF	Yes	NA	No	D
9	M	Neg	TN+LN	59	7	265.0	1162.0	T4aN1bM1	2	Dec/35	0	NDF	Yes	Yes	No	A (1.86)
10	M	Met918Thr	LN	58	3.5	NA	NA	T4amN1bM0	>10	0	0	NA	Yes	Yes	Yes	D
11	F	Neg	LN	46	10	418.2	88512.0	T4aN1bM1	17	Dec/29	0	NDF	Yes	NA	No	D
12	F	Ala883Phe	TN+LN	59	4.5	16493.0	158014.0	T4amN1bM1	>10	Dec/32	0	NDF	Yes	Yes	Yes	D
13	F	Met918Thr	TN	53	2.1	68.9	97706.0	T2N1aM1	0	Dec/12	0	NDF	No	NA	No	D
14	F	Met918Thr	TN	49	4	86.6	833.0	T2N1bM0	18	143	0	NDF	No	Yes	No	D
15	F	Cys634Arg	TN	67	3.5	179.4	81.7	T2N0M0	0	141	0	NDF	No	Yes	No	D
16	F	Neg	TN	60	5.5	261.0	Undetect.	T3N1aM0	1	79	79	DF	No	No	No	D
17	F	Met918Thr	TN+LN	26	3	NA	NA	T2mN1bMX	18	NA	NA	NA	No	Yes	Yes	D
18	M	Cys620Ser	TN	31	1.8	7.8	Undetect.	T1N0M0	0	124	124	DF	No	No	No	D
19	M	Ala883Phe	TN+LN	55	5	110.5	33800.0	T3mN1bM1	10	Dec/85	0	NDF	No	Yes	Yes	D
20	F	Neg	TN	72	8.5	12.3	823.0	T3mN1bM0	12	49	0	NDF	No	NA	Yes	D
21	M	Met918Thr	TN+LN	49	2	141.0	3999.0	T4aN1bM0	7	56	0	NDF	Yes	NA	No	A (1.65)
22	M	Met918Thr	TN	71	4.5	8525.0	14584.0	T4amN1bM0	37	11	0	NDF	Yes	Yes	Yes	D
23	F	Neg	TN	27	NA	NA	78.3	TXN1bM0	NA	252	0	NDF	NA	NA	NA	D
24	M	Cys620Arg	TN	70	4.5	6.5	Undetect.	T3N1bM0	3	61	61	DF	No	NA	NA	A (1.93)
25	F	Met918Thr	TN+LN	66	4	Undetect.	Undetect.	T2N1bM0	3	70	70	DF	No	No	No	D
26^b^	F	Neg	TN	47	3.5	Undetect.	Undetect.	T2N0M0	0	72	72	DF	No	No	No	A (1.95)
27	F	Met918Thr	TN	50	1.7	84.8	25.9	T4aN1aM0	6	45	0	NDF	Yes	Yes	NA	D
28	F	Neg	TN	61	3	Undetect.	Undetect.	T2N0M0	0	48	48	DF	No	No	No	D
29	M	Met918Thr	LN	43	2.5	60307.0	103334.0	T2N1bM1	30	41	0	NDF	No	No	No	D
30	F	Cys634Arg	TN	71	1.6	Undetect.	Undetect.	T1N0M0	0	46	46	DF	No	NA	No	D
31	F	Cys630Arg	TN	67	4.3	Undetect.	Undetect.	T3N0M0	0	113	113	DF	No	No	No	A (1.97)
32	F	Met918Thr	LN	60	0.8	1234.0	1205.0	T1N1bM0	8	103	0	NDF	No	NA	No	D
33	F	Neg	TN	61	3.5	47.5	Undetect.	T2N0M0	0	177	177	DF	No	No	No	D
34	F	Cys630Arg	TN	62	2.5	10.4	Undetect.	T2N0M0	0	178	178	DF	No	No	No	D
35	M	Met918Thr	TN	67	3	8.5	15725.0	T2mN1bM0	8	146	0	NDF	No	No	Yes	D
36	M	Met918Thr	TN	29	5	36.4	Undetect.	T3N0M0	0	135	135	DF	No	Yes	No	A (1.83)
37	F	Neg	TN+LN	77	8	2582.0	17732.0	T3N1bM1	8	119	0	NDF	No	Yes	No	D
38	F	Met918Thr	TN	57	2.5	8.8	10.6	T2N0M0	0	132	132	NDF	No	No	No	D
39	F	Met918Thr	TN	32	1.7	Undetect.	Undetect.	T1N0M0	0	121	121	DF	No	No	No	D
40	F	Met918Thr	LN	51	2.3	1970.0	9953.0	T4amN1bM1	10	21	0	NDF	Yes	No	Yes	A (1.86)
41^b^	F	Neg	TN	27	3	Undetect.	Undetect.	T2N0M0	0	100	100	DF	No	Yes	No	D
42	F	Neg	NA	82	1.2	Undetect.	Undetect.	T1N0M0	0	75	75	DF	No	No	No	D
43	M	Met918Thr	LN	29	1.5	36720.0	245000.0	T1mN1bM1	4	Dec/100	0	NDF	No	No	Yes	D
44	M	Neg	TN	40	2	268.0	46.9	T2N1bM0	3	178	0	NDF	No	Yes	No	D
45	M	Val882Val+Ala883Phe	LN	35	10	34342.0	42694.0	T4aN1bM1	27	Dec/94	0	NDF	Yes	No	No	D
46	F	Cys634Tyr	TN	46	2	Undetect.	Undetect.	T1N0M0	0	123	123	DF	No	No	No	D
47	M	Met918Thr	TN+LN	44	3.5	7400.0	20884.0	T2N1bM1	>12	101	0	NDF	No	NA	No	D
48	F	Neg	TN	42	0.9	Undetect.	Undetect.	T1N0M0	0	16	16	DF	No	No	No	D
49	F	Neg	TN+LN	46	1	89.0	128.0	T3mN1bM0	10	18	0	NDF	Yes	Yes	Yes	D
50	M	Neg	TN+LN	61	5	13762.0	33687.0	T4amN1bM0	16	18	0	NDF	Yes	Yes	Yes	D
51	F	Cys618Arg	TN+LN	61	10	550.0	NA	T4aN1bM1	4	Dec/4	0	NDF	Yes	NA	No	D

Abbreviations: A=aneuploid; CT=calcitonin; D=diploid; Dec=deceased from MTC; DF=disease free; Extragl. ext.=extraglandular extension; F=female; LN=lymph node; M=male; MTC=medullary thyroid carcinomas; Multif.=multifocality; NA=not available; NDF=non-disease free; Neg=negative for *RET* mutations; No.=number; Post-oper.=post-operative; *RET*=*RE*arranged during *T*ransfection; TN=thyroid nodule; TNM=Tumour-Node-Metastases; Undetect.=undetectable; Vasc. inv.=vascular invasion.

a (the biggest).

b Partial thyroidectomy.

**Table 2:** *RET* mutations identified in sporadic MTC cases

**Exon**	**Type of alteration**	**Number of patients**	**% of patients carrying a *RET* somatic mutation**
10	TGC>CGC (Cys618Arg)^a^	1	9.1%
	TGC>CGC (Cys620Arg)	1	
	TGC>TCC (Cys620Ser)	1	
11	TGC>CGC (Cys630Arg)	2	21.2%
	**TGC>GGC (Cys630Gly)**	1	
	TGC>CGC (Cys634Arg)	2	
	TGC>TAC (Cys634Tyr)	1	
	**c.1881del18+ TGC>TGT (Cys634Cys)**	1	
15	GCT>TTT(Ala883Phe)+ GTA>GTT (Val882Val)	1	9.1%
	GCT>TTT (Ala883Phe)	2	
16	ATG>ACG (Met918Thr)	20	60.6%

Abbreviations: MTC=medullary thyroid carcinomas; *RET*=*RE*arranged during *T*ransfection.

a This mutation was in the hemizygous status.

Mutations were present only in tumour DNA.

The two novel *RET* proto-oncogene variants are represented in bold.

**Table 3:** Clinical and pathological characteristics of patients with Met918Thr and Ala883Phe (group 1) *vs* other *RET* mutations (group 2) or no mutation (group 3) in sporadic MTC

**Characteristics**	**Group 1 Met918Thr and Ala883Phe *RET* mutation (*n*=23, 45.1%)**	**Group 2 Other *RET* mutation (*n*=10, 19.6%)**	**Group 3 No *RET* mutation (*n*=18, 35.3%)**	***P*-value**
*Sex*				0.2175^a^
Female	52.2% (12/23)	70.0% (7/10)	77.8% (14/18)	
Male	47.8% (11/23)	30.0% (3/10)	22.2% (4/18)	
				
*MTC presentation*				0.099^b^
Thyroid nodule	39.1% (9/23)	90.0% (9/10)	64.7% (11/17)	
Lymph node	26.1% (6/23)	0.0% (0/10)	11.8% (2/17)	
Thyroid nodule and lymph node	34.8% (8/23)	10.0% (1/10)	23.5% (4/17)	
*Age at surgery (years), mean±s.e.m.*	50.00±2.80	59.60±4.19	55.78±3.85	0.1835^c^
*Tumour size (cm), mean±s.e.m.*	3.59±0.42	3.77±0.84	4.68±0.81	0.4240^c^
				
*Post-operative serum calcitonin*				0.132^b^
Undetectable	9.5% (2/21)	30.0% (3/10)	37.5% (6/16)	
Detectable	90.5% (19/21)	70.0% (7/10)	62.5% (10/16)	
				
*Serum calcitonin at last control*				***0.0119*** a
Undetectable	14.3% (3/21)	66.7% (6/9)	47.1% (8/17)	
Detectable	85.7% (18/21)	33.3% (3/9)	52.9% (9/17)	
				
*T categories*				0.664^b^
T1	13.0% (3/23)	40.0% (4/10)	17.6% (3/17)	
T2	39.1% (9/23)	20.0% (2/10)	29.4% (5/17)	
T3	13.0% (3/23)	20.0% (2/10)	23.5% (4/17)	
T4	34.8% (8/23)	20.0% (2/10)	29.4% (5/17)	
				
*T categories grouping*				0.6944^a^
T1–T3	65.2% (15/23)	80.0% (8/10)	70.6% (12/17)	
T4	34.8% (8/23)	20.0% (2/10)	29.4% (5/17)	
				
*Lymph node metastases*				***0.0051*** a
N1	87.0% (20/23)	30.0% (3/10)	61.1% (11/18)	
N0	13.0% (3/23)	70.0% (7/10)	38.9% (7/18)	
				
*Distant metastases*				0.0588^a^
M1	45.5% (10/22)	11.1% (1/9)	16.7% (3/18)	
M0	54.5% (12/22)	88.9% (8/9)	83.3% (15/18)	
*Presence of extraglandular extension*	36.4% (8/22)	20.0% (2/10)	35.3% (6/17)	0.6314^a^
*Presence of vascular invasion*	52.9% (9/17)	33.3% (2/6)	50.0% (7/14)	0.823^b^
*Presence of multifocality*	40.9% (9/22)	0.0% (0/9)	17.6% (3/17)	***0.037*** b
				
*Ploidy pattern*				0.758^b^
Diploid	87.0% (20/23)	80.0% (8/10)	88.9% (16/18)	
Aneuploid	13.0% (3/23)	20.0% (2/10)	11.1% (2/18)	
				
*Stage*				0.065^b^
I	4.3% (1/23)	30.0% (3/10)	16.7% (3/18)	
II	4.3% (1/23)	20.0% (2/10)	22.2% (4/18)	
III	4.3% (1/23)	10.0% (1/10)	5.6% (1/18)	
IV	87.0% (20/23)	40.0% (4/10)	55.6% (10/18)	
				
*Stage grouping*				***0.0145*** a
I–III	13.0% (3/23)	60.0% (6/10)	44.4% (8/18)	
IV	87.0% (20/23)	40.0% (4/10)	55.6% (10/18)	
*Number of positive lymph nodes, mean±s.e.m.*	11.48±2.09	1.20±0.63	5.24±1.64	***0.0017*** d
*Follow-up (months), mean±s.e.m.*	91.8±15.1	101.3±16.9	90.8±17.6	0.9180^c^
				
*Status at last control*				***0.0242*** a
Disease free	14.3% (3/21)	60.0% (6/10)	44.4% (8/18)	
Non-disease free	85.7% (18/21)	40.0% (4/10)	55.6% (10/18)	

Abbreviations: MTC=medullary thyroid carcinomas; *RET*=*RE*arranged during *T*ransfection.

a *χ*^2^-test,

b Fisher's exact test,

c One-way analysis of variance and

d Kruskal–Wallis test.

*P-*values in *italics* and bold are statistically significant.
